# Burden of foodborne diseases: think global, act local

**DOI:** 10.1016/j.cofs.2021.01.006

**Published:** 2021-06

**Authors:** Sara M Pires, Binyam N Desta, Lapo Mughini-Gras, Blandina T Mmbaga, Olanrewaju E Fayemi, Elsa M Salvador, Tesfaye Gobena, Shannon E Majowicz, Tine Hald, Peter S Hoejskov, Yuki Minato, Brecht Devleesschauwer

**Affiliations:** 1 National Food Institute, Technical University of Denmark, Lyngby, Denmark; 2 School of Public Health and Health Systems, University of Waterloo, Waterloo, Canada; 3 National Institute for Public Health and the Environment, Bilthoven, The Netherlands; 4 Utrecht University, Institute for Risk Assessment Sciences, Utrecht, The Netherlands; 5 Kilimanjaro Clinical Research Institute-Kilimanjaro Christian Medical Centre, Moshi, Tanzania; 6 Kilimanjaro Christian Medical University College, Moshi, Tanzania; 7 Department of Biological Sciences, Mountain Top University, Ibafo, Ogun State, Nigeria; 8 Department of Biological Sciences, Faculty of Sciences, Eduardo Mondlane University, Maputo, Mozambique; 9 College of Health and Medical Science, Haramaya University, Ethiopia; 10 World Health Organization, Copenhagen, Denmark; 11 World Health Organization, Geneva, Switzerland; 12 Department of Epidemiology and Public Health, Sciensano, Brussels, Belgium; 13 Department of Veterinary Public Health and Food Safety, Ghent University, Merelbeke, Belgium

## Abstract

National burden of foodborne disease (FBD) studies are essential to establish food safety as a public health priority, rank diseases, and inform interventions. In recent years, various countries have taken steps to implement them. Despite progress, the current burden of disease landscape remains scattered, and researchers struggle to translate findings to input for policy. We describe the current knowledge base on burden of FBDs, highlight examples of well-established studies, and how results have been used for decision-making. We discuss challenges in estimating burden of FBD in low-resource settings, and the experience and opportunities deriving from a large-scale research project in these settings. Lastly, we highlight the role of international organizations and initiatives in supporting countries to develop capacity and conduct studies.

## Highlights

• National burden of disease studies are needed to define food safety priorities.
• Initiatives to support capacity building and collaboration are emerging.
• Novel methodologies provide opportunities to improve data collection for disease burden estimation in low-resource settings.

**Current Opinion in Food Science** 2021, **39**:152–159

This review comes from a themed issue on **Food microbiology**

Edited by **Anderson Sant’Ana**

For complete overview of the section, please refer to the article collection, “Food microbiology”

Available online 1st February 2021

**https://doi.org/10.1016/j.cofs.2021.01.006**

2214-7993/© 2021 The Author(s). Published by Elsevier Ltd. This is an open access article under the CC BY license (http://creativecommons.org/licenses/by/4.0/).

## Introduction

Foodborne diseases (FBD) still cause a substantial public health, economic and social burden worldwide. Recognizing the need to measure the burden and distribution of FBD and encourage evidence-informed policies, in 2015 the World Health Organization (WHO) reported the first estimates of global and regional disease burden due to 31 foodborne hazards [1]. Results showed that, each year, 1 out of 10 people get ill from food contaminated with microbial or chemical agents, resulting in 600 million illnesses, 420 000 deaths and the loss of 33 million healthy years of life globally [2^•^]. While these estimates were crucial to raise awareness, they were the product of an enormous research initiative that faced substantial data gaps. Importantly, they did not offer the precision needed to identify priorities at the national level, and were not always able to make use of all data resources available. Precise national disease burden estimates are essential to identify the most important diseases and hazards in a country, as well as the foods contributing the most to these diseases and the interventions needed to effectively prevent them.

In recent years, various countries have recognized the need for studies of the national burden of FBDs, and have taken steps to implement them. Despite progress, these represent mostly high income countries in a few regions of the world; pre-COVID, many other countries still lacked political commitment, technical and financial resources, and data to estimate the burden of FBDs, and we anticipate these barriers will increase as a result of the pandemic. Furthermore, the current burden of disease landscape remains scattered, and researchers struggle to translate their findings to useable input for decision makers.

Promoting national burden of FBD studies now relies on a combination of factors. First, the utility of burden of disease estimates for risk ranking, priority setting and efficient allocation of resources for food safety needs to be communicated to the appropriate target audiences, particularly policy makers. Second, there is a need for harmonizing methodologies, sharing data and data collection approaches, and building technical capacity in countries and globally; these are important for comparison of estimates across diseases, countries and regions, and for facilitating the study implementation process. Third, efforts to leverage on novel technologies and possibilities for decreasing the costs and facilitating widespread data collection for burden of disease studies, particularly in low-income and middle-income countries (LMIC), should be made to ensure that all regions of the world can have increasingly complete and robust burden of FBD estimates.

The aim of this paper is to contribute to this process. We describe the current knowledge base on burden of FBDs, and highlight examples of well-established national studies, as well as the utility of their results for policy making and establishing public health priorities. Next, we discuss the main challenges to estimating burden of FBD in low-resource settings, and the experience and opportunities deriving from a large-scale research project in these settings. Lastly, we highlight the role of international organizations, particularly the WHO, in supporting countries to develop capacity and conduct country-level burden of disease studies. The contents described were compiled in the context of a workshop held at the World Public Health Conference, October 2020 [3].

## Current landscape of national burden of foodborne disease studies

The concept of burden of disease was developed in the 1990s by the Harvard School of Public Health, the World Bank and the WHO to describe death and loss of health due to diseases, injuries and risk factors for all regions of the world [4]. While burden of disease can be expressed using various indicators, such as incidence, mortality, societal costs and summary measures of population health, this study introduced a new metric, the Disability-Adjusted Life Year (DALY), which combines information on morbidity and mortality caused by diseases. The DALY is now the most widely used public health metric for burden of disease studies, and the key measure in the Global Burden of Disease (GBD) studies [5^•^].

The first estimates of the burden of foodborne diseases in DALYs were published in 2000, measuring the health burden of a single foodborne pathogen in the Netherlands [6]. Other studies followed in Europe and beyond, using the DALY metric to measure the burden of country-specific single pathogens or a few of foodborne pathogens [7, 8, 9, 10, 11, 12, 13, 14]. In 2015, the WHO produced the first global DALY estimates of the burden of FBD [1]. A few other countries have established burden of FBD studies after that, publishing either routine estimates or ad-hoc reports or articles with estimates for specific pathogens and years. These largely focus on microbiological agents and include mostly high-income countries, such as the Netherlands [15,16^•^], Japan [17], Denmark [18,19], Belgium [20], and the United States of America [21]. Initiatives in low-income countries have taken the first steps to address knowledge gaps. As examples, [22] estimated of the incidence of illness of FBDs from syndromic surveillance data in Rwanda, and the Caribbean Burden of Illness Study estimated the prevalence and incidence of acute gastroenteritis and specific foodborne pathogens in nine countries [23]. These are first steps to national burden of disease studies. Evidence on the burden of individual foodborne chemicals in some countries has also been published [24, 25, 26]. Again, these studies were conducted in high-income countries, where data are more abundant, but the disease burden may be lower when compared to LMIC.

Even if available evidence illustrates that LMICs bear a higher burden than high-income countries, the emphasis is on the data gaps. For example, the WHO’s sub-regional DALY estimates for LMICs majorly relied on imputation, where data available from some countries were used to predict data missing from others, as data were scarce in these countries [1,27^•^,^28^, ^29^, ^30^, ^31^]. Given the target of the WHO’s global DALY estimates and learning lessons from the few nationwide studies, estimation of the burden of nation-based foodborne diseases would be suitable for informing interventions and policy.

### WHO-FERG’s country studies efforts

The Foodborne Disease Burden Epidemiology Reference Group (FERG) was established by WHO in 2007 to estimate the global and regional burden of FBDs (across the six WHO regions) [32]. Other aims of FERG were to strengthen the capacity of countries to assess their burden of FBD, and to increase the number of countries that have undertaken such a study. Activities of FERG to promote national studies involved capacity-building and promotion of the use of information on burden of disease in setting evidence-informed policies. The FERG Country Studies Task Force (CSTF) developed a suite of tools and resources to support national studies. Pilot studies were conducted in Albania, Japan, Thailand and Uganda [31] and provided important practical lessons. In particular, data gaps impeded DALY calculations in several occasions. These gaps included information needed to assign the etiology for syndromes, such as acute gastrointestinal disease, and data on the incidence of diseases caused by some hazards.

The pilot studies also highlighted the need for engagement of stakeholders that can provide access to national data, including public and private data sources. In some countries, private hospitals provide a significant proportion of health care, and may not adhere to the same reporting requirements as public hospitals. Engagement with private hospitals and other facilities may need to be specifically addressed to provide a complete picture of the incidence of FBDs. Data on foodborne hazards may be gathered from primary producers and the food industry, but economic implications, particularly for trade, may constitute a barrier for sharing such data, which therefore requires careful handling.

The CSTF concluded that the use of findings from national burden of FBD studies is facilitated when stakeholders with a role and interest in food safety, such as governmental institutions, academia, and decision-makers (Ministry of Health, Ministry of Agriculture, Ministry of Environment, food safety authorities, public health agencies) — work closely with the study team from the earliest stages. They can be involved in early and continuous efforts to incorporate knowledge translation and risk communication to the relevant audiences. A detailed description of all country studies and support materials developed by the CSTF has been published [31].

## Filling-in data gaps to estimate burden of foodborne diseases where data are scarce

LMICs, in particular from Africa, bear the highest burden of FBDs [2^•^,27^•^]. In the African region where the FBD burden is the highest, the 31 foodborne hazards included in the WHO estimates of the global burden of FBD have been estimated to cause 1200–1300 DALYs per 100 000 inhabitants in 2010, compared to 35–711 in other regions. Nearly 70% of the burden is due to diarrheal disease agents, particularly to non-typhoidal *Salmonella* (including invasive salmonellosis), and Enteropathogenic and Enterotoxigenic *Escherichia coli*. Other important agents included *Vibrio cholerae* and *Taenia solium* [1]. However, because research and disease surveillance data from Africa are limited, these estimates are subject to uncertainty. The main challenge to estimating burden of FBD in Africa is lack of data, particularly on the incidence of FBDs in the population. This limited availability is caused by various factors, such as the lack of capacity to generate, compile and analyse data, limited political commitment to strengthen surveillance systems, limited understanding of the benefits of burden of disease studies and a focus on selected notifiable priority diseases.

To address this, a multi-country project launched in 2019 in Ethiopia, Mozambique, Nigeria, and Tanzania, aims to estimate the burden of, and strengthen surveillance systems for, FBDs in Africa [33]. The team is conducting a population-based survey (to estimate incidence of diarrhea in the community), a systematic literature review (to estimate proportions of diarrheal disease caused by different agents), and an active review of available FBD reports (to estimate the extent of underreporting in existing surveillance). Together, these findings will provide more accurate estimates of the burden of FBDs in African contexts. The tools and lessons from this large-scale project can be extrapolated to other countries and regions where the burden is high, but data are scarce. The data collection tools being developed will be available to be adapted for other settings. Other highlights include applying leadership roles, delegation of duties and project tasks, setting milestones, regular meetings, and risk-mitigation plans. Because of their local knowledge on FBDs and of the functioning of institutions, the leading role of experts in this project helps to reduce hurdles. The project has also adapted existing data collection tools such as questionnaires and survey study design for use across the diverse African study populations. It is engaging stakeholders, including policy-makers, who will use its research outputs, by involving them at all stages of the project. This integrated knowledge translation approach is translatable to other settings.

## Using novel methodologies to support burden of disease estimates

An apparent challenge to estimating burden of FBDs, particularly in LMICs, where laboratory capacity and surveillance systems are limited, is obtaining valid estimates of etiology proportions of cases. A commonly used method is systematic review of studies reporting pathogen isolation in diarrhea cases [34, 35, 36]. However, studies often differ in design, population, timeframe, and pathogens included, hampering extrapolation to the target population.

The above-mentioned project is exploring a novel approach for estimating diarrhea etiology proportions in urban and rural populations in the four African countries [37]. It analyses sewage samples using short-read next-generation sequencing (NGS) to determine abundance of genes that can be mapped to specific bacterial genera, providing an estimate of the relative abundance of pathogens in each sample. By combining results with the diarrheal incidence estimated in parallel, pathogen-specific incidence will be estimated and compared with incidence estimates from the traditional approach.

The application of NGS to human sewage has great potential for surveillance of FBDs, particularly in resource-poor settings where laboratory capacity for bacterial isolation is limited. First, NGS is a ‘one method takes all’ approach, as it is based on detection of RNA/DNA, a language common across pathogens. Second, it is culture-independent, allowing for real-time data generation and standardized sharing. Finally, few samples are needed to survey large populations for several pathogens at the same time. Thus, surveillance based on NGS applied to sewage may prove to be an indirect measure of incidence. Although it will not provide an estimate for the true incidence in the population, it will increase our understanding of the burden and, as such, be a proxy and novel way of ranking diseases. However, the sustainability of the application of NGS in resource-poor settings remains an issue and will require directing resources for building capacity.

## From science to policy: the experience of well-established burden of foodborne disease studies

Estimates of the burden of FBDs are useful to prioritize food safety policy and allocate resources to where food safety risks are highest. The experiences of established studies and of their mechanisms of translation of evidence into policy can provide guidance and suggest processes for other national studies. Here we focus on two countries that have been at the forefront of burden of FBD estimation: the Netherlands and Denmark.

### The Netherlands

Burden of FBD estimates have been published every year in the Netherlands since 2008 [14,38]. The Dutch Ministry of Health mandates the Dutch National Institute for Public Health and the Environment (RIVM) to provide annual updates of the number of illnesses, disease burden and cost-of-illness caused by an agreed-upon panel of 14 enteric pathogens mainly transmitted by food. The disease burden is expressed in DALYs. The cost-of-illness related to these pathogens is estimated in euros (€), and includes healthcare costs, the costs for the patient, family and caregivers (e.g. travel and external care expenses), and costs in other sectors, for example productivity losses [14,39]. Demographic data, as well as data on mortality, live births and stillbirths, are obtained from the Dutch Central Bureau of Statistics. The incidence of infections by pathogen is obtained from various surveillance systems. For instance, data on pathogens like *Listeria monocytogenes*, *Shiga*-toxin producing *E. coli* (STEC) O157 and hepatitis A virus, which are notifiable, are obtained from case notifications and the laboratory surveillance system, which has national coverage. For pathogens that are not (mandatorily) notifiable, such as *Campylobacter*, *Salmonella*, *Cryptosporidium*, norovirus and rotavirus, data are obtained from case notifications or laboratory surveillance based on networks of sentinel diagnostic laboratories. The collected data are corrected for geographical coverage of the surveillance system and for under reporting, to obtain an estimate of the incidence. Moreover, using different approaches to source attribution, the estimated DALYs and cost estimates are attributed to five major transmission pathways (i.e. food, environment, direct animal contact, human-human transmission, travel) and 11 food groups within the foodborne pathway. The RIVM has regularly published the burden and cost estimates on its website and in reports, for example [39].

The most recent estimates, for 2019, show that the 14 pathogens are cumulatively responsible for about 11 000 DALYs and € 423 million [40]. The share apportioned to foodborne transmission is estimated at 4200 DALYs and € 174 million. The largest foodborne burden at population level was caused by *Campylobacter,* followed by *Toxoplasma gondii* and norovirus. Regarding other foodborne bacteria, *Salmonella* ranked second after *Campylobacter* spp. Perinatal listeriosis and congenital toxoplasmosis were the diseases with the highest individual burden. The pathogens causing the largest costs were norovirus, rotavirus, *Staphylococcus aureus*, and *Campylobacter*. However, the average cost per case was largest for perinatal listeriosis (€ 291 000/case). Healthcare costs accounted for 21% of the total costs for the 14 pathogens, patient and family costs for 2%, and the costs in other sectors accounted for 77%. About 41% of the foodborne burden was associated with meat, that is, poultry, pork, beef and lamb, which caused 33% of all food-related fatal cases, indicating that the pathogens associated with these foods are responsible for the most severe infections.

Year after year, these national estimates have provided vital insights for policy-making as to guide strategies, such as establishing process hygiene criterion for *Campylobacter* on broiler meat [41]. Yet, they play an even more vital role in resource allocation, such as funding for research and other activities on specific pathogens or conditions that appear to have a higher burden. Burden and cost estimates also enable policy-makers and the scientific community to monitor trends and generate scientific hypotheses. For instance, although the disease burden for *Campylobacter* had continually decreased since 2010, it slightly increased in 2018 and 2019, suggesting a beginning of a reversal of the trend. This calls for more research, such as studies focusing on hygiene measures at primary production, performance of surveillance and diagnostics, risk factor analyses, as well as genomics of circulating strains, to understand the underlying causes.

### Denmark

The first Danish burden of FBD study was published in 2014, and has been growing with new hazards and data being added at different points in time [18,42,43]. The study currently includes microbiological and chemical hazards commonly present in foods.

The most recent estimates for microbiological agents are from 2017 and cover seven pathogens that are mostly transmitted through foods: *Campylobacter*, *Salmonella*, STEC, norovirus, *Yersinia enterocolitica*, *L. monocytogenes*, and *Toxoplasma gondii*.

In 2017, *Campylobacter* caused the highest burden of disease, more than threefold higher than the second highest ranked pathogen, *Salmonella*. *Listeria* and *Yersinia* followed in the ranking. The burden of congenital toxoplasmosis was lower than most of the investigated diseases but was borne by a low number of cases in the population.

The ranking of foodborne pathogens varied substantially when based on reported cases, estimated incidence, and burden of disease estimates. The total estimated incidence was highest for norovirus, but this agent ranked sixth when focusing on foodborne burden measured in DALYs. These differences illustrate the importance of estimating burden of disease in DALYs, particularly when the purpose is to compare across diseases with very diverse severity and duration.

Most of the foodborne pathogens can also be transmitted through non-foodborne routes, and the study partitions to overall burden of disease to foods, and, for some pathogens, links with source attribution estimates for specific foods. For attribution to main transmission routes, the burden estimates were linked with the source attribution proportions estimated for the European sub-region that includes Denmark by the FERG’s expert elicitation [44]. *Campylobacter* still led the ranking when excluding DALYs attributable to non-foodborne routes of exposure, but the ranking of some of the other pathogens (particularly norovirus) changed.

While these estimates were not initially requested by the Danish Veterinary and Food Administration (DVFA), they are now used to identify priorities and to determine efforts for further surveillance and interventions, to inform allocation of resources for research, or to promote discussions of the public health relevance of pathogens in the country. For example, the DVFA formed pathogen-specific interest groups that gather experts from public health, food and animal surveillance to discuss priorities and define needs to inform policy. Among these, the *Campylobacter* Interest Group, formed in 2017, meets approximately four times a year to discuss how stakeholders can make the best use of the data generated and the knowledge gained from research and surveillance data for a better monitoring of *Campylobacter* in food and humans. The interest group also contributes information on research, including routes of infection, infection dynamics, and genetic methods to distinguish campylobacter from different sources. The DVFA has also initiated several activities in the parasitic area, including a risk profile of foodborne parasites in Denmark, and communication of information to the consumer.

## Capacity building and support from international organizations

In line with global and regional strategies and based on country-support plans and biennial collaboration agreements with Member States, WHO, in collaboration with its partners and collaborating centres, provides technical assistance to countries to strengthen national food safety systems. This includes technical assistance to generate, collect and analyse food safety evidence, including information about the burden of FBDs. For instance, in Vietnam, identification of priority pathogens for FBDs informed the expansion of the emerging disease surveillance system and strengthening IHR core capacities for surveillance in the country, and in Albania, a pilot study on the national burden of FBDs provided important input to the process of reorganizing and strengthening the national food safety system [45,46].

WHO has a critical role in providing evidence for action to improve food safety and to support member states to effectively collect, analyse, report and use data on FBDs [47]. The WHO estimates of the global burden of FBDs were the first ever attempt to describe the magnitude of such To monitor trends in the global burden of FBDs and provide an updated basis for food safety policy development, WHO has started the process of updating the 2010 estimates. This includes a review of the methodology and epidemiological data, identification of technical gaps and priorities for research, and establishment of task forces and other means through which scientific and technical matters related to the burden of FBDs can be addressed.

WHO is also accelerating its efforts and support to member states to estimate the national burden of FBDs. This is done through technical assistance and development of guidance to assess the burden of FBDs caused by microbiological agents at national level. The guidance includes a complete picture of the requirements, enabling factors, challenges and opportunities to estimate the burden of FBDs, and of the steps for deriving the estimates.

WHO promotes the use of harmonized methodologies for estimating foodborne disease burden across countries. A harmonized approach provides an opportunity for countries to compare their disease burden with the one of other countries and is essential for experiences to be shared and food safety policy to be improved.

In addition to the WHO, other international organizations are playing a pivotal role in quantifying national and global (foodborne) disease burden. Most notably, the Institute for Health Metrics and Evaluation (IHME) is responsible for the GBD study, currently generating estimates for 369 diseases and injuries and 87 risk factors in 204 countries and territories [48]. While food safety is not included as a separate entity, the GBD study does cover several individual FBDs. In collaboration with IHME, the Global Burden of Animal Diseases (GBADs) programme, which is being developed by a group of international collaborators led by the University of Liverpool, United Kingdom, has recently been established. The programme aims to strengthen and complete the GBD estimates of FBD burden and to leverage these to evaluate food safety [49]. These initiatives will further promote the inclusion of food safety on the global health agenda.

## Steps forward

The ground for promoting national burden of FBDs studies is being paved. To take the awareness of the usefulness of burden of disease studies to guide food safety interventions in the direction of actual implementation of studies, international organizations, local authorities and the scientific community will have specific and multiple roles.

Burden of foodborne disease studies make the basis for informed risk management decisions. This is a key priority of the forthcoming Global Food Safety Strategy developed by WHO. The strategy will serve as a strategic framework to guide action of governments to strengthening national food safety control systems. The upcoming WHO guidance to estimate burden of FBD in countries and planned activities for capacity building will play a crucial part in encouraging countries to develop and launch their national studies. While these will need to be accompanied by resources to implement and run the project, they may motivate institutions and/or research groups to start with small-scale projects that have the potential to be extended over time.

Along with the support from international organizations (such as WHO), networks of experts with experience in burden of disease studies are of value for technical support, knowledge sharing and harmonization of methods. One of these is the European Burden of Disease Network (COST Action CA18218, www.burden-eu.net), which is already contributing within its European members and associated partners from other regions. The sustainability and expansion of this and similar efforts can be an important contribution for an increase in the number of national burden of FBD studies.

Furthermore, the development and dissemination of new approaches and data collection tools, particularly for LMIC, will be valuable to overcome one of the biggest challenges faced so far — the data scarcity faced in many countries globally. Innovative tools have the potential of being of faster, wider and cheaper application, thus reducing the disparity of data availability between high income countries and LMIC.

Burden of disease can be expressed using various indicators, such as incidence, mortality, societal costs and summary measures of population health. The DALY is recognized as the ultimate summary measure for quantifying the population health impact of foodborne diseases. While estimating DALYs is an aspirational goal, any step towards it is valuable. Estimates of incidence and mortality can also be used to rank and compare the public health impact of FBDs and should be encouraged to take as a first step in burden of disease studies.

## Declaration of interests

The authors declare that they have no known competing financial interests or personal relationships that could have appeared to influence the work reported in this paper.

## References and recommended reading

Papers of particular interest, published within the period of review, have been highlighted as:

• of special interest
